# Endoscopically visible steam pop during high-energy laser pulmonary vein ablation

**DOI:** 10.1007/s12471-015-0711-8

**Published:** 2015-05-19

**Authors:** P. Gal, J.J.J Smit, A. Elvan

**Affiliations:** Department of Cardiology, Isala Klinieken, Dr. van Heesweg 2, 8025 AB Zwolle, The Netherlands

## Abstract

A 58-year-old woman with atrial fibrillation underwent laser balloon ablation at our centre. During 12 W ablation in the left superior pulmonary vein, a sudden steam pop was witnessed with displacement of the balloon catheter. Visualisation of the pulmonary vein antrum showed a red discolouration at the last ablation site.

The endoscopically assisted laser balloon ablation system (EAS) is a relatively novel technique that is being used to perform pulmonary vein isolation (PVI) in the treatment of atrial fibrillation [1]. The EAS consists of a flexible, compliant balloon for sustained wall contact and a power adjustable laser beam for ablation independent of tissue contact.

A 58-year-old woman underwent PVI with the EAS due to drug-refractory, symptomatic and paroxysmal atrial fibrillation. During 12 W ablation at the antrum of the left superior pulmonary vein (LSPV), a sudden steam pop was witnessed, with displacement of the EAS catheter (Fig. 1). Visualisation of the LSPV antrum showed a red discolouration, most likely a haematoma in the antral wall of the LSPV, at the last ablation site. A successful PVI was performed; the red discolouration was still present after 1 h. The patient did not develop symptoms related to the steam pop and echocardiography did not reveal any abnormalities.

**Fig. 1:**
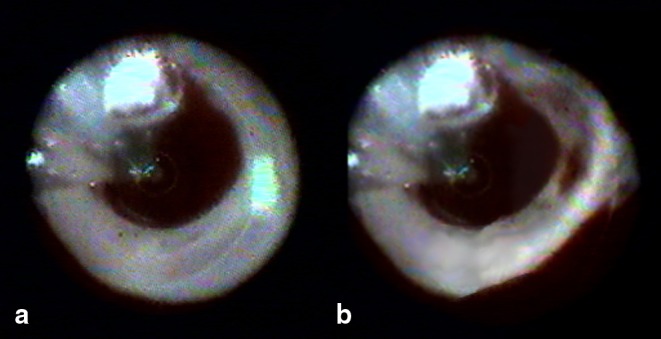
Witnessed steam pop during endoscopically assisted ablation. Panel **a** displays the fifth ablation site in the left superior pulmonary vein (LSPV) where the steam pop occurred. The *white* ring of exposed tissue is a sign of optimal catheter-wall contact. Panel **b** displays the LSPV antrum directly after the steam pop. Note the *red* discolouration which was not present in panel **a**

Steam pops are caused by overheating of myocardial tissue, exceeding 100 ℃, and are preceded by a shift in impedance levels, which cannot be measured with the EAS. Higher energy settings and higher contact force are known to increase the risk of steam pops. Steam pops can lead to tissue disruption and cardiac perforation [2]. However, steam pops appear to be a rare complication with reduced EAS energy settings, which we mostly used in 50 EAS patients, in whom no steam pops were observed [3].

## Funding

None.

## Conflict of interest

None declared.
